# Retraction: α-bisabolol enhances radiotherapy-induced apoptosis in endometrial cancer cells by reducing the effect of XIAP on inhibiting caspase-3

**DOI:** 10.1042/BSR-2019-0696_RET

**Published:** 2022-08-31

**Authors:** Dongmei Fang, Hongxin Wang, Min Li, Wenwen Wei

**Keywords:** α-bisabolol, apoptosis, endometrial cancer, radiotherapy, XIAP/caspase-3 pathway

This Retraction follows an Expression of Concern relating to this article previously published by Portland Press. This article is being retracted from *Bioscience Reports* at the request of the authors following receipt of a notification from a reader alerting the Editorial Office to western blot bands that appear in both this paper and another paper by different authors (doi: 10.1186/s12935-019-0985-9). The authors wish to retract the article. The Editor-in-Chief and Editorial Board agree with the retraction.

